# Round the Hospitals

**Published:** 1920-09-18

**Authors:** 


### ROUND THE HOSPITALS.

The new scale of pay for Army nurses does not mark so great a rise as might have been anticipated considering the enormous rise in prices during the last six years. Sisters, who before the war received £50, now begin at £75, rising by yearly increments of £5 to £85. Staff nurses, who formerly began at £40, now begin at £60. As regards matrons, the service has expanded, and now comprises much variety of administrative duty under this term. The Army matron formerly began at £75, and rose by annual increments of £10 to £150. Under the new scale she begins at £115 and rises by annual increments of £10 to £185. This is a great improvement. The matrons of military families' hospitals, however, still begin at £75 a year, plus charge pay of £20 a year up to thirty beds and £30 a year for over thirty beds. Their maximum salary is £105 for the smaller hospitals and £115 for the larger. As regards retired pay, the matrons of military families' hospitals, other than Aldershot, are on the same footing as sisters, and receive a maximum of £75 a year. Matrons are granted seven weeks' leave in the year, sisters six weeks, and staff nurses five weeks, in each case an additional week compared with the period before the war.

The contrast between the charming pictures of nurses at play appearing last week in the illustrated dailies and the fulminations in an evening paper against the nurses' "living death" and "purgatory" are not a little funny, and calculated to puzzle the public. Whether we watch the winning competitor in a shooting contest between nurses at the Prince of Wales' General Hospital, Tottenham, or take glimpses at the lively lawn tennis competition at Marylebone for the *Nursing Times'* Cup between nurses from various London hospitals, we are getting a saner, more representative view of the nurse's lot than is revealed in the flaming headlines of our contemporary. Truth to tell, there are very bad moments in the lives of all nurses, for the forces of disease and death they are set to encounter in their patients' interests are more grim than lie in the path of the teacher or typist. But to represent these desperate contingencies as making up the entire lot and outlook of a nurse is as misleading as it would be to paint it all tennis and *couleur de rose*. The obvious remedy for nurses subjected to abominable conditions of work and quarters is to resign and state their reasons for doing so. Wherever this course is courageously followed a deadly blow is struck at nursing abuses.

Well-planned courses of lectures, commencing this month, are advertised by the National Association for the Prevention of Infant Mortality and for the Welfare of Infancy. Elementary lectures on infant care will be given at 6 p.m. every Monday, beginning September 27, at Morley Hall, George Street, Hanover Square. They are intended for lay people, infant-welfare workers, teachers, and mothers, and form a course of preparation for the elementary certificate of the Association. The fee for the course of twelve lectures is 7s. 6d. Advanced lectures on infant care, specially intended for crèche nurses and probationers, will be held every Thursday from September 30 at 7.30 in the Essex Hall, Essex Street, Strand. This course is intended to prepare for the crèche nurse's certificate now instituted by the Association. Students must be qualified by twelve months' training at an approved day nursery and gain a certificate for proficiency in practical work there. The above forms the second of the two courses necessary for qualification. Voluntary workers in day nurseries are also invited to attend the lectures, of which the fee for the whole course is 10s. Courses of lectures on the same subjects have also been arranged to take place at the University College, Nottingham. All information can be obtained from the Secretary, 4/5 Tavistock Square, W.C.

The College of Nursing is in the possession of a suitable building for a residential club for College members, in the vicinity of the present headquarters. It is anticipated that the alterations and improvements necessary will take about three months, so that the opening of the club should take place early in the New Year.

Mr. Graham Campbell, delivered a considered judgment at Bow Street Police Court on September 8 when he dismissed Mrs. Florence Maud Johnson's appeal against the cancellation by the London County Council of her registration as a person carrying on a "twilight sleep" lying-in home in Courthill Road, Lewisham. After summing up the evidence which had been given with reference to the man Tattersall, said to have been adopted as a son by Mrs. Johnson and her late husband, he stated he was reluctantly forced to believe that Mrs. Johnson was a person of bad character. He was of opinion that the equipment of the premises was unsuitable for a lying-in home, and he therefore came to the conclusion that on both grounds the order of the County Council must be confirmed.

### ROUND THE HOSPITALS—(*continued*).

He completely exonerated Dr. Summerskill from the charge that he showed bias against Mrs. Johnson. The magistrate made an order for fifty guineas costs, it being stated that the London County Council had incurred actual out-of-pocket expenses for a sum considerably over £100.

---

Some anxiety, we learn, is being felt about the nursing sisters in Mesopotamia. A considerable number of Englishwomen were in residence in the area now disturbed by the Arab rising. Every effort was doubtless made at the outset to send them away from the zone of hostilities, but nurses are always reluctant to be banished from the hospitals, and it is possible some are still on duty in the Euphrates district.

---

An animated debate took place at the quarterly meeting of the Governors of the Cumberland Infirmary on September 8, when the chair was occupied by Dr. Barnes. It arose out of a motion by Dr. Lediard that a copy of the report of Sir Napier Burnett, issued after inquiry by him into the management of the infirmary, be forthwith sent to each Governor of the institution. The inquiry conducted by Sir Napier Burnett arose out of a round robin signed by twenty-seven nurses and probationers last February, which brought to light grievances which, in Dr. Lediard's words, "ought never to have existed in a well-managed hospital." Sir Napier Burnett made a careful investigation, interviewed nurses and members of the staff, and sent in a report dealing with the grievances of the nurses and the management of the infirmary. Strong objections were raised by Mr. Allen Hodgson and the Dean of Carlisle to issuing the report to the Governors, on the ground that it would be prejudicial to the infirmary to do so, that it dealt with various strictly personal matters, and reflected necessarily upon the conduct of various individuals connected with the hospital, though it decided that the conduct of no individual was such as to cause their removal. In the end it was agreed that the report should be open to the perusal of any Governor who was interested in it, at the infirmary. It transpired during the debate that the Infirmary Committee do not propose to carry out all Sir Napier Burnett's proposals, "owing to the financial condition of the hospital."

---

The Isle-of-Wight County Council has been caught napping over its nursing arrangements. They engaged a new county superintendent nurse, Miss G. E. Bailey, at the beginning of this year, but lost her at once when she discovered that, contrary to what she has been given to understand, the Committee refused to provide a bicycle for her use. Her immediate resignation woke up the authorities, who in advertising the post again raised the salary by £25 to cover cost of uniform, cycle and laundry. Dr. J. P. Walker, medical officer of health, enters a stern protest in his report against the parsimonious treatment meted out to district nurses and midwives by their employers as contrasted with that accorded to school teachers. The rate of pay, he declares, is little more than half that of a certificated assistant teacher in an elementary school. The hours are long, there are seven days' work a week, no regular meal-times, fewer holidays, no gratuity after a life of great usefulness to the community, heavy laundry expenses and a uniform to provide, no pension either for old age or broken health. The case against the County Authorities is pretty bad, but so used is the public to under-pay nurses that we fear it will pass unnoticed. Dr. Walker does public service in officially declaring "Such a state of affairs should be altered without delay."

**ROUND THE HOSPITALS—*(continued)*.**

At a garden fête held at Clumber on September 2 in aid of the Notts Nursing Federation the Duchess of Newcastle stated that a scheme was on foot for starting a training-centre for village nurses, and will be supported by the County Council if sufficient funds are forthcoming. There should be no difficulty in providing such a home in a centre so fortunate as to number the Dukeries in its midst, but more efficient publicity is required to bring the needs of the Federation and its aims into prominence. Every county ought to have its own training-home for midwives, and be able to provide such supplementary instruction in general nursing as will enable their pupils to be useful in the ordinary sick-room of the village home. Until fully certificated nurses show a greater disposition to take up district nursing as a career village nurses will continue to be indispensable. They constitute a large proportion of the practising midwives registered by the Central Midwives Board.

The sum of £310 rewarded the supporters of the Victoria Nursing Association in their street collections made in Portsmouth on September 4. The Mayoress who took her station at the Town Hall, collected £24, and the Victoria nurses themselves, at two different stations, £45. Much has been said against street collections, but it remains true that they form the most expeditious and agreeable way of attracting small sums for objects dependent on popular support, and they do, at any rate, succeed in enlisting the sympathies of that elusive individual "the man in the street." It would have been a thankless and laborious task to collect as much as £300 by a house-to-house visitation.

Dyspeptic patients are a sad trial to their relations, and do not always fare very well, if rumour speak true, in hospitals, in pay wards, and in nursing homes. A very useful aid in the difficult task of luring them to enjoy food is afforded by "Cookery for Dyspeptics" (Mr. Edwin Arnold, 2s. 6d.), written by the late Dr. George Herschell. It originally formed an appendix to Dr. Herschell's well-known "Text-book of Indigestion," and the value of the hints there provided has been so widely recognised that a great desire has been expressed to have them in a more compact form. Beginning at the lowest rung of the ladder—egg-water—the manual takes the nurse agreeably through meat juices and jellies, soups, gruels, and purées, food in pultaceous form, semi-solid preparations such as soufflés, pâtés, and purées of meat, game, and chicken, to solid food, where the principles of roasting, boiling, and frying are discussed tersely but with effect. We demur to some of the doctor's recipes for cooking vegetables. The use of stock can only disguise the delicate flavour of these choice articles of diet.

Novel contests were held under the Order of St. John of Jerusalem at Pontyclun recently, when the Countess of Plymouth presented a new shield and medals in connection with the Pontypridd competitions in first aid, and a silver bowl and medals for the nursing associations. Two teams competed for the home nursing contests, and the rose bowl was secured by Mrs. D. Tuckett's No. 1 Llanharren team. A great impetus has been given to Red Cross work since the creation of the Grand Priory for Wales, and it is interesting to find that the local colliery companies recognise the importance of what is being done and have presented beautiful trophies for the successful teams.

A capital sale has been held at Bridlington Red Cross Convalescent Home for Soldiers and Sailors, under the inspiration of Miss Brooks, the matron, and her capable staff of nurses. Lady Carisbrooke opened the proceedings, supported by Lord and Lady Nunburnholme, and under a large attendance of buyers the sale proved an uncommon success. The amount realised was £250.

A very sad incident is reported from Margate, where a little patient, Patrick O'Reilly, suffering from tubercular disease of the spine, fell out of bed at the Princess Mary Hospital in one of the open-air verandahs in such a way as to fracture his spine at the base of the neck. The little boy, who was nearly five years old, had been under treatment since last December, suffering from tuberculosis of the spine in the region of the neck, and had been kept in a recumbent position by means of a strap until recently, when, as he was progressing towards recovery, the strap was removed for a short time every day. During one of these intervals he reached over unperceived by the nurse in charge of the ward, and fell out of bed, striking himself in such a way that he fractured the diseased part. The coroner, in summing up, declared there was no sort of evidence of neglect or carelessness in the case, and recorded a verdict that the child died from a fractured spine resulting from a fall from the bed. He spoke warmly "from personal knowledge" of the nursing and general efficiency at the hospital, as did also the mother of the patient.

The Americans certainly have a genius for nomenclature. Who would not be an "administrative institutional dietitian" rather than a housekeeper? Unfortunately it is possible to become an "A.I.D." it appears by the age of twenty-two, and institutions look askance at the finished product. We are not sure, however, that in securing the title of "psychiatric nurses" for women who have been trained in the observation of brain and nerve disease, our sisters over the seas have not brought light into a dark place. The word "mental" is ridiculously inept as a description of either a nurse or a patient, and used indiscriminately of both betrays its absurdity. The psychiatric nurse is needed more and more, and we rejoice to notice the dawning theory that this science should make part of ordinary training.

